# Plastid genome evolution in tribe Desmodieae (Fabaceae: Papilionoideae)

**DOI:** 10.1371/journal.pone.0218743

**Published:** 2019-06-24

**Authors:** Dong-Pil Jin, In-Su Choi, Byoung-Hee Choi

**Affiliations:** Department of Biological Sciences, Inha University, Michuhol-gu, Incheon, Republic of Korea; Indiana University Bloomington, UNITED STATES

## Abstract

Recent plastid genome (plastome) studies of legumes (family Fabaceae) have shown that this family has undergone multiple atypical plastome evolutions from each of the major clades. The tribe Desmodieae belongs to the Phaseoloids, an important but systematically puzzling clade within Fabaceae. In this study, we investigated the plastome evolution of Desmodieae and analyzed its phylogenetic signaling. We sequenced six complete plastomes from representative members of Desmodieae and from its putative sister Phaseoloid genus *Mucuna*. Those genomes contain 128 genes and range in size from 148,450 to 153,826 bp. Analyses of gene and intron content revealed similar characters among the members of Desmodieae and *Mucuna*. However, there were also several distinct characters identified. The loss of the *rpl2* intron was a feature shared between Desmodieae and *Mucuna*, whereas the loss of the *rps12* intron was specific to Desmodieae. Likewise, gene loss of *rps16* was observed in *Mucuna* but not in Desmodieae. Substantial sequence variation of *ycf4* was detected from all the sequenced plastomes, but pseudogenization was restricted to the genus *Desmodium*. Comparative analysis of gene order revealed a distinct plastome conformation of Desmodieae compared with other Phaseoloid legumes, i.e., an inversion of an approximately 1.5-kb gene cluster (*trnD-GUC*, *trnY-GUA*, and *trnE-UUC*). The inversion breakpoint suggests that this event was mediated by the recombination of an 11-bp repeat motif. A phylogenetic analysis based on the plastome-scale data set found the tribe Desmodieae is a highly supported monophyletic group nested within the paraphyletic Phaseoleae, as has been found in previous phylogenetic studies. Two subtribes (Desmodiinae and Lespedezinae) of Desmodieae were also supported as monophyletic groups. Within the subtribe Lespedezinae, *Lespedeza* is closer to *Kummerowia* than *Campylotropis*.

## Introduction

**Fig 1:**
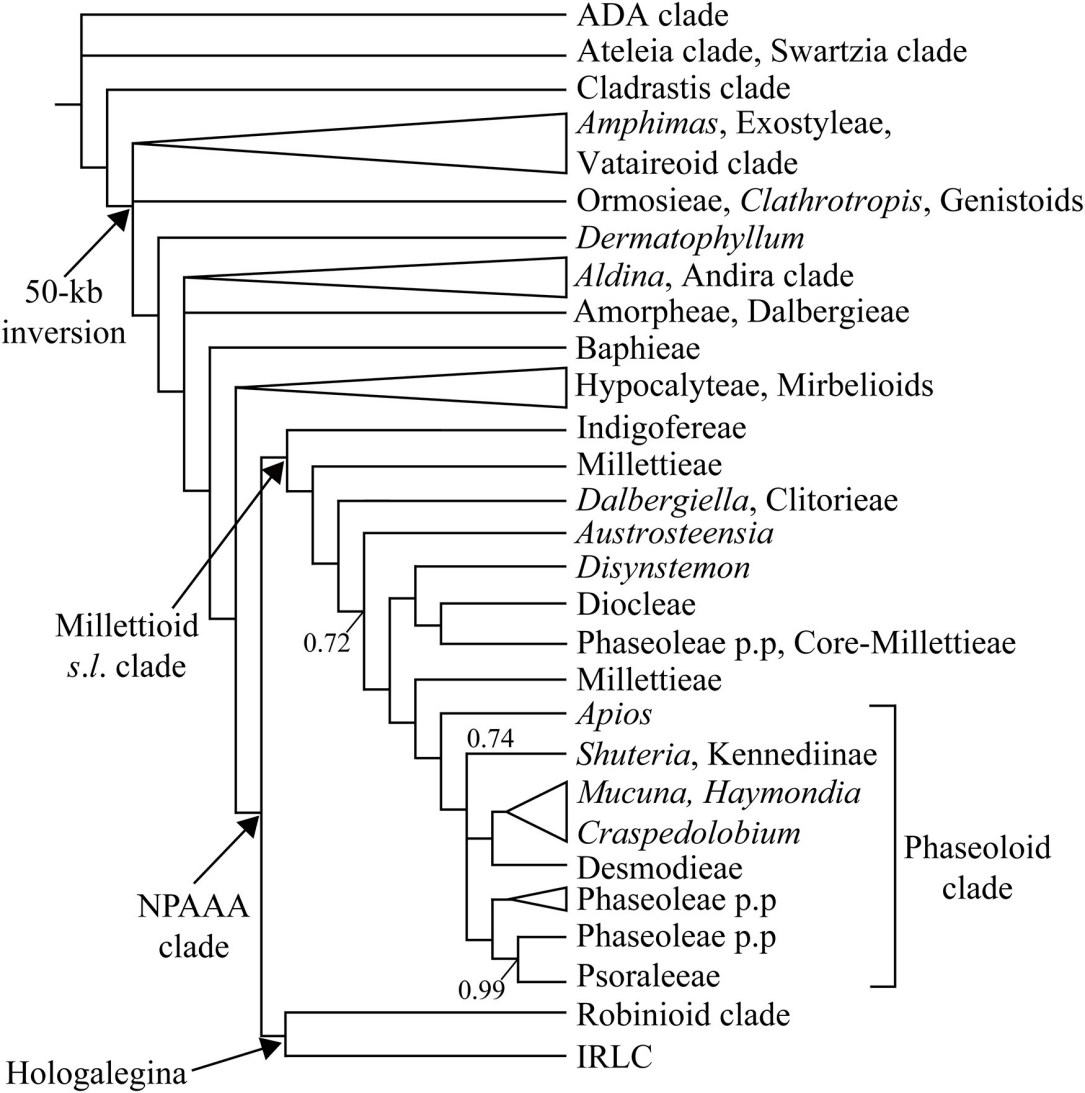
Phylogeny of Papilionoideae modified from Legume Phylogeny Working Group [1–2].

Fabaceae (Leguminosae), the third largest family within the angiosperms, includes 770 genera and over 19,500 species [1]. Three well-known subfamilies–Caesalpinioideae, Mimosoideae, and Papilionoideae–have recently been reclassified into six subfamilies–Caesalpinioideae, Cercidoideae, Detarioideae, Dialioideae, Duparquetioideae, and Papilionoideae [2]. The Phaseoloid clade is one lineage within Papilionoideae, which comprises the Phaseoleae sensu lato (s.l.) clade, Desmodieae, and Psoraleeae [3]. Many species belonging to this clade are utilized as a source for food, ornamental foliage, and medicine. This clade displays a complex phylogenetic relationship among and within tribes [1, 4]. Tribe Desmodieae and Psoraleeae are monophyletic groups that are nested within the paraphyletic Phaseoleae s.l. group [1, 4] (Fig 1).

The tribe Desmodieae comprises 32 genera [5]. Although these plants mainly grow in tropical and warm-temperate regions, some members are found in the cool-temperate and boreal regions of North America [5–6]. Species of this tribe usually occur in the form of herbs or shrubs, and rarely as trees [5]. Their fruits are either loments (a loment consists of a single carpel that disarticulates into single-seeded segments when ripe) or legumes (fruits composed of a single article) [7]. This tribe has traditionally been split into three subtribes: Bryinae, Desmodiinae, and Lespedezinae [7]. Among these three, Bryinae has now been placed in Dalbergieae s.l. as a result of molecular phylogenetics studies [8–10]. The latter two subtribes are still considered as part of Desmodieae [5]. Ohashi [5] used *rbcL* phylogeny [11] and morphological traits to place the groups DESMODIUM, PHYLLODIUM, and LESPEDEZA within the Desmodieae tribe. The DESMODIUM and PHYLLODIUM groups that belong to the subtribe Desmodiinae, include 17 (e.g. *Desmodium* Desv. and *Hylodesmum* H. Ohashi & R.R. Mill) and 12 (e.g. *Ohwia* H. Ohashi, *Phyllodium* Desv., and *Ougeinia* Benth.) genera, respectively. The LESPEDEZA group corresponds with subtribe Lespedezinae. This system has been confirmed via subsequent phylogenetic tree that used two chloroplast DNA regions (*rbcL*, *psbA*-*trnH*) [12], albeit genus *Ougeinia* was placed within DESMODIUM group. Within the Lespedezinae, the relationship among the three genera–*Campylotropis* Bunge, *Kummerowia* Schindl., and *Lespedeza* Michx.–has been debated based on their inflorescence traits [13–14] or floral structures [15]. Data from phylogenetic studies based on chloroplast DNA and nuclear ribosomal DNA internal transcribed space (nrDNA ITS) of *Lespedeza* [16–17] have supported the opinion of Nemoto and Ohashi [14–15], i.e. *Lespedeza* is closer with *Kummerowia* than *Campylotropis*.

The plastid genome (plastome) is a source of numerous characters for phylogenetic as well as comparative genomics investigations [18]. For the plastomes of seed plants, their shared conformations, i.e., a quadripartite structure [large single copy (LSC), small single copy (SSC), and pair of inverted repeats (IRs)], gene content, and gene order, have been accepted as their usual characters [19]. Because the typical plastome has a conserved nature, the high number of copies and uni-parental inheritance have been acknowledged as excellent characters for use as molecular markers [12, 19]. Molecular phylogenies, based on complete plastome sequences, have become common and are now used to resolve many phylogenetically puzzling relationships. However, extensive studies have also found that atypical genome structures and gene contents frequently occur among several distantly related families [20]. Among fully photosynthetic angiosperms, the variation in their genome structures is largely attributable to the expansion and contraction of IRs, the most extreme examples being the complete deletion of one copy (e.g., the IR-lacking clade of Fabaceae) and the expansion to an entire single-copy region shown from the genus *Asarum* L. within Aristolochiaceae [21–22]. Genome rearrangements via large inversions and reductions in gene numbers have been reported from Campanulaceae [23–24], Geraniaceae [25–27], and Oleaceae [28]. Therefore, these atypical characters have provided unprecedented insight into plastome evolution and important traits that can help decipher phylogenetic relationships.

For legumes in the family Fabaceae, Doyle et al. [29–30] and Bailey et al. [8] have conducted studies with polymerase chain reactions (PCRs) and probe hybridization to reveal lineage-specific inversions (a 50-kb inversion for most of the papilionoid clade and a 78-kb inversion for Phaseoleae subtribe Phaseolinae) as well as gene or intron losses (e.g., the loss of the *rpl2* intron and *ycf4* in a core member of Desmodieae). Jansen et al. [19] revealed a *rps12* intron loss in Desmodieae and most of IRLC (inverted repeat-lacking clade). More recently, complete plastome studies have re-validated those early findings and elucidated other interesting and atypical evolutions. These include large novel inversions from subfamily Cercidoideae [31–32] and Papilionoideae [33–36]; an accelerated substitution rate [37–38]; and multiple gene and intron losses [19, 32, 35–36]. Some of these characters are lineage-specific and can assist in resolving phylogenetic relationships at different ranking levels, such as the 24-kb inversion for Sophoreae [36]. In contrast, some characters are shared among distantly related taxa, making them unreliable for circumscribing taxa. Those include the *rps16* loss and 36-kb inversion for distantly related legume lineages [35–36].

Several molecular studies have surveyed the phylogenetic relationship of Desmodieae with related tribes (e.g. [8, 12, 19]). Those examinations, however, were conducted using restricted taxonomic sampling and/or individual genes, thereby limiting detailed discussions. Although more recent extensive plastome research on Fabaceae has greatly increased our understanding of its evolution, the available resources are still insufficient when considering the vast diversity within that family. In particular, the complete plastome of Desmodieae in the Phaseoloid clade has never been analyzed and the exact status of introns (*rpl2* and *rps12*) and *ycf4* losses from the tribe have not been surveyed by genome sequencing. Therefore, the purpose of our research described here was to provide important insight into legume plastome genomics and systematics by sequencing and analyzing the plastome of some species of Desmodieae. Our specific aims included: 1) sequencing of six complete plastomes for Desmodieae and one from its putative sister genus *Mucuna* Adans., 2) analyzing plastome evolution, and 3) presenting a discussion about the phylogenetics of Desmodieae among Phaseoloid legumes based on plastome sequences and characters.

## Materials and methods

### Ethics statement

The plant species we sampled in Korea and in Japan are neither endangered nor protected. We did not collect any plant from protected areas.

### Plant sampling

We designed our sampling strategy to represent Desmodieae genera and the genus *Mucuna*, which is a sister group of this tribe [1–4]. It included six species of Desmodieae [*Lespedeza maritima* Nakai, *Kummerowia striata* (Thunb.) Schindl, *Campylotropis macrocarpa* (Bunge) Rehder, *Desmodium heterocarpon* (L.) DC., *Hylodesmum podocarpum* (DC.) H. Ohashi & R.R. Mill subsp. *podocarpum*, and *Ohwia caudata* (Thunb.) H. Ohashi], and *M*. *macrocarpa* Wall. These species were sampled in order to represent the main groups recognized in this tribe [5]. *Campylotropis*, *Kummerowia*, and *Lespedeza* represented subtribe Lespedezinae and the LESPEDEZA group while the remaining three genera covered subtribe Desmodiinae. *Desmodium* and *Hylodesmum* were defined as the DESMODIUM group while *Ohwia* was representative of the PHYLLODIUM group. Leaves were collected and then preserved with silica gel. Sampling information is shown in S1 Table.

### DNA sequencing and genome assembly

Genomic DNAs of the seven species were extracted from the silica-dried leaves with a Qiagen DNeasy Kit (QIAGEN, Seoul, Korea). The extracted genomic DNAs were visualized in 2% agarose gels by electrophoresis, and their quality and quantity were assessed with a NanoDrop ND-1000 (NanoDrop Technologies, Wilmington, DE, USA). The extracted DNA (*Lespedeza maritima*, 300 ng; all others, 150 ng) was fragmented to 500 bp with a Covaris S220 (Covaris, Woburn, Massachusetts, USA). A TruSeq Nano DNA Library Preparation Kit (Illumina, San Diego, California, USA) was used for library preparation after sequencing on the Illumina MiSeq platform at Life Is Art of Science (LAS; Gimpo, Korea; http://lascience.co.kr/).

For each species, we produced 2,982,620 to 10,758,454 paired-end reads (301 bp for each) (S1 Table). Low-quality reads were removed by Trimmomatic 0.32 [39]. The plastome sequences were assembled following a process we reported previously [36]. First, the paired-end reads of *L*. *maritima* were mapped onto the plastomes of the Phaseoloids: *Apios americana* Medik. (NC_025909), *Glycine soja* Siebold & Zucc. (NC_022868), *Phaseolus vulgaris* L. (EU196765), and *Vigna radiata* (L.) R. Wilczek (NC_013843) with “Medium-Low Sensitivity” settings in Geneious 7.1.8 (Biomatters Ltd., Auckland, NZ). Using those mapped reads, we then conducted the *de novo* assembly under Medium-Low Sensitivity in Geneious. Afterward, we confirmed all of the assemblies by read depths for the paired-end sequence data. Some regions showing low coverage of reads (<50) were re-checked by PCR, as were the plastome junctions, i.e., LSCs, SSCs, and IRs. The PCR products were treated with an MG PCR Purification Kit (MGmed, Seoul, Korea), and sequenced at Macrogen (Seoul, Korea). Subsequently, the paired-end reads of the six remaining species were mapped to the plastome of *L*. *maritima*, and the assembled plastomes were confirmed by the method described above. The process of read mapping and assembly was repeated using assembled contigs as references if the first assembly produce more than one plastome contigs. All primers were designed with Primer3 software [40].

### Genome annotation, alignments, and visualization

The plastid genes for all seven tested species were annotated using Geneious 7.1.8, based on the annotation of *Glycine max* (L.) Merr. (NC_007942). This process was implemented when nucleotide sequences of the plastid genes for tested species showed over 90% of similarity with reference genome. Some protein-coding genes were manually identified by considering their start and stop codons. The tRNAs were confirmed by searching in tRNAscan-SE [41] and by comparing with reference data. Genome maps were drawn with OGDraw [42]. We deposited our complete plastome sequences in the GenBank database (https://www.ncbi.nlm.nih.gov/) (MG867566–MG867572).

### Analysis of gene/intron losses and genome rearrangement

Comparative analyses for intron, gene, and genome rearrangement included the seven completed plastomes of this study, and six completed plastome data of Phaseoleae legumes (S2 Table). Four genes (*rpl2*, *rps12*, *rps16*, and *ycf4*) were extracted from each plastome. Intron losses and pseudogenization for each sequence were aligned using the default parameters of MUSCLE [43], and manually edited. The entire plastome was aligned using progressive Mauve 2.3.1 [44] with default settings to check for a genome rearrangement event. The order of genes in the cluster *trnD*-*GUC*, *trnY*-*GUA*, and *trnE*-*UUC* was compared between Desmodieae and the Phaseoloid legumes by sequence alignment as described above. Expansion or contraction of the IR region was investigated and visualized by IRscope [45].

### Phylogenetic analysis

Data from ten additional plastomes from GenBank were used to construct a phylogenetic tree for Desmodieae (S2 Table). Millettioids and Indigofereae plastomes were used as outgroups, because these two taxa were the sister groups to Phaseoloids in previous phylogenetic studies [1–2]. In all, we selected 67 plastid protein-coding genes that are conserved among those taxa (S3 Table). After combining gene sequences according to each taxon, we aligned them with MUSCLE under default parameters. Poorly aligned regions were either manually refined or deleted using Geneious 7.1.8. The final sequence alignment is available at “Supporting information” (S1 Fasta File). To construct the tree with a Maximum Likelihood (ML) analysis, we chose a nucleotide substitution model throughout jModelTest 2.1.6 [46]. For this process, total 88 substitution models were compared along with a gamma distribution of site heterogeneity; followed by GTR + I + G being selected according to the Akaike Information criterion (AIC). The aligned data set was employed to construct an ML tree through RAxML [47] with 1,000 replicates.

## Results

### Plastid genome sequences and contents

**Fig 2:**
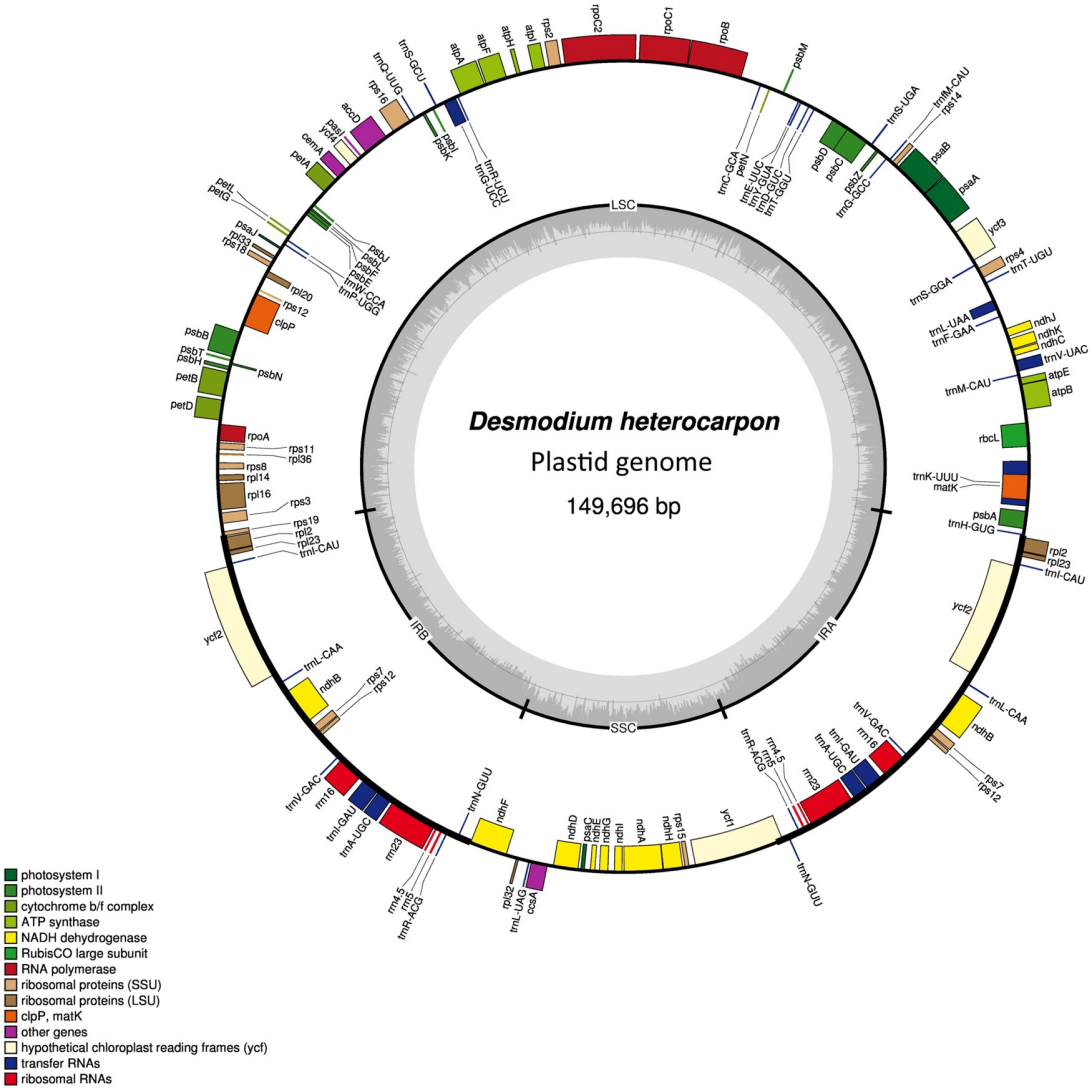
Plastid genome map of *Desmodium heterocarpon*, as representative of tribe Desmodieae. Genes on outside of outer circle are transcribed in clockwise direction; those on inside of outer circle are transcribed in counterclockwise direction. Colored rectangles indicate functional genes, with categories shown on bottom left. Gray scale in inner circle indicates GC content of plastid genome.

The plastid genome maps for six species from tribe Desmodieae and one from *Mucuna* are illustrated in Fig 2, S1 Fig and Table 1. *Desmodium heterocarpon* was the representative of Desmodieae. Plastome maps for the remaining species are shown in S1 Fig. The complete plastome of Desmodieae members ranges in length from 148,450 to 150,249 bp, and consists of an LSC (81,942–83,241 bp), an SSC (18,159–18,939 bp), and two IRs (each 23,720–24,264 bp). The plastome of *Mucuna* (153,826 bp) is larger than that of the Desmodieae species due to expansion by intergenic spacers (IGSs). Each genome harbors 128 genes, including 83 protein-coding genes, eight rRNA genes, and 37 tRNA genes (S4 Table). Approximately 49.7 to 52.5% of the total genome consists of protein-coding region, while the remaining 47.5 to 50.3% is composed of tRNA, rRNA, introns, and IGSs. The AT and GC contents are 64.8 to 65.1% and 34.9 to 35.2%, respectively. The plastome of *M*. *macrocarpa* is similar to that of the Desmodieae members based on genome features such as gene contents and length.

**Table 1:** Genomic information for species analyzed from tribe Desmodieae and genus *Mucuna*.

Species	Accession number	Total genome size (bp)	Length of LSC(bp)	Length of SSC(bp)	Length of IR(bp)	GC content (%)	No. of genes	Pseudogenized gene	SSC-IRa Junction	Inversion type*
Tribe Desmodieae subtribe Desmodiinae										
* Desmodium heterocarpon*	MG867567	149,696	82,967	18,441	24,144	35.2	128	*ycf4*	*ycf1* runs from SSC to IRa	A
* Hylodesmum podocarpum*	MG867568	149,564	83,125	18,159	24,140	35.2	128	-	*ycf1* runs from SSC to IRa	A
* Ohwia caudata*	MG867572	150,249	83,241	18,480	24,264	35.1	128	-	*ycf1* runs from SSC to IRa	A
Tribe Desmodieae subtribe Lespedezinae										
* Campylotropis macrocarpa*	MG867566	148,814	82,566	18,808	23,720	34.9	128	-	*ycf1* located only at SSC	B
* Kummerowia striata*	MG867569	148,450	81,942	18,860	23,824	35.0	128	-	*ycf1* located only at SSC	B
* Lespedeza maritima*	MG867570	149,022	82,429	18,939	23,827	35.0	128	-	*ycf1* located only at SSC	B
Tribe Phaseoleae										
* Mucuna macrocarpa*	MG867571	153,826	85,873	18,357	24,798	35.3	128	*rps16*	*ycf1* runs from SSC to IRa	-

*A, inversion of colinear block (*trnE*-*Y*-*D*); B, inversion of colinear block with 500-bp deletion upstream of *psbM*.

For these seven species, we observed the loss of *infA* and *rpl22* that is typical of all legumes (S4 Table). The introns of *rpl2* and *rps12* were absent from all six Desmodieae species while *Mucuna* lost the *rpl2* intron but retained the *rps12* intron (S2 Fig). Although the sequence of *ycf4* was highly variable (S3 Fig), the open reading frame (ORF) was intact for all but *Desmodium*. In particular, *ycf4* from *D*. *heterocarpon* lost its start codon through substitution and showed abundant internal stop codons due to mutations in the nucleotide sequence. In *M*. *macrocarpa*, *rps16* had a 70-bp deletion in exon 2 that resulted in a severe frame shift of amino acids (S4 Fig). Consequently, we deemed *ycf4* of *D*. *heterocarpon* and *rps16* of *M*. *macrocarpa* to be pseudogenes.

### Genomic rearrangement

**Fig 3:**
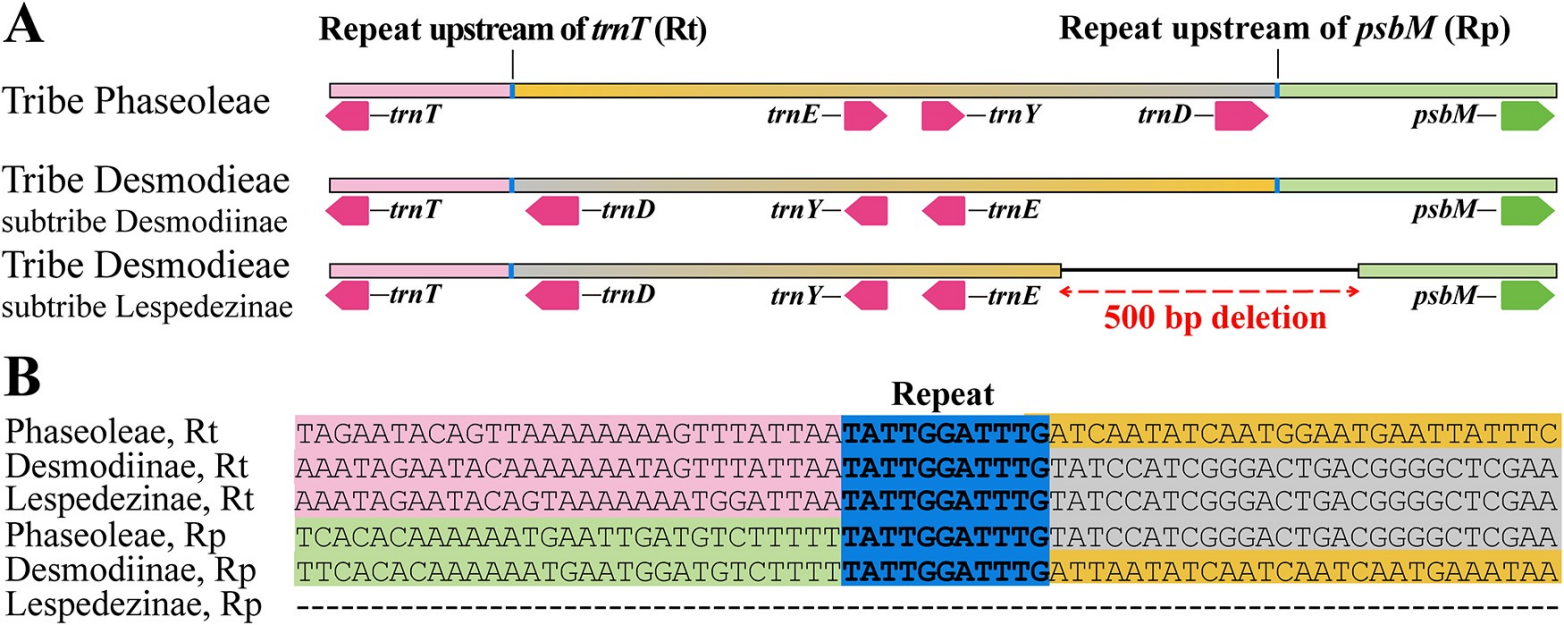
Desmodieae-specific inversion of gene cluster *trnE-UUC*, *trnY-GUA*, and *trnD-GUC*, mediated by the 11-bp inverted repeats at inversion break points. (A) Comparisons of inversion for each tribe and subtribe. Nucleotide sequences were simplified in narrow rectangles; same-colored rectangles indicate similar sequence. The 11-bp repeats (blue bars) coincide with break points of inversion. (B) Aligned repeat motifs located upstream of *trnT-GGU* (Rt) and upstream of *psbM* (Rp). Rp sequences were reversely aligned.

We examined structural changes in the Desmodieae and *Mucuna* plastomes by comparing them with other legumes. A 50-kb inversion in the LSC, common to most members of Papilionoideae, was shared among all the six Desmodieae species and *M*. *macrocarpa* (Fig 2 and S1 Fig). In addition, six plastomes exhibited a novel inversion (ca. 1.5-kb) of gene cluster *trnE*-*UUC*, *trnY*-*GUA*, and *trnD*-*GUC* specific to Desmodieae, located in the LSC (Fig 3). This inversion was situated between *trnT*-*GGU* and *psbM*. Among Phaseoloids, the typical arrangement of the five genes was *trnT*-*GGU*—*trnE*-*UUC*—*trnY*-*GUA*—*trnD*-*GUC*—*psbM*. However, members of Desmodieae had an inverted arrangement of *trnT*-*GGU*—*trnD*-*GUC*—*trnY*-*GUA*—*trnE*-*UUC*—*psbM*. The break point of this inversion coincided with a pair of 11-bp inverted repeats located on either side of *trnE*-*Y*-*D* (TATTGGATTTG and CAAATCCAATA) (Fig 3). However, this break point was absent from subtribe Lespedezinae (*Campylotropis macrocarpa*, *Kummerowia striata*, and *Lespedeza maritima*) because of a 500-bp deletion from the IGS of *trnE*-*UUC* and *psbM*.

**Fig 4:**
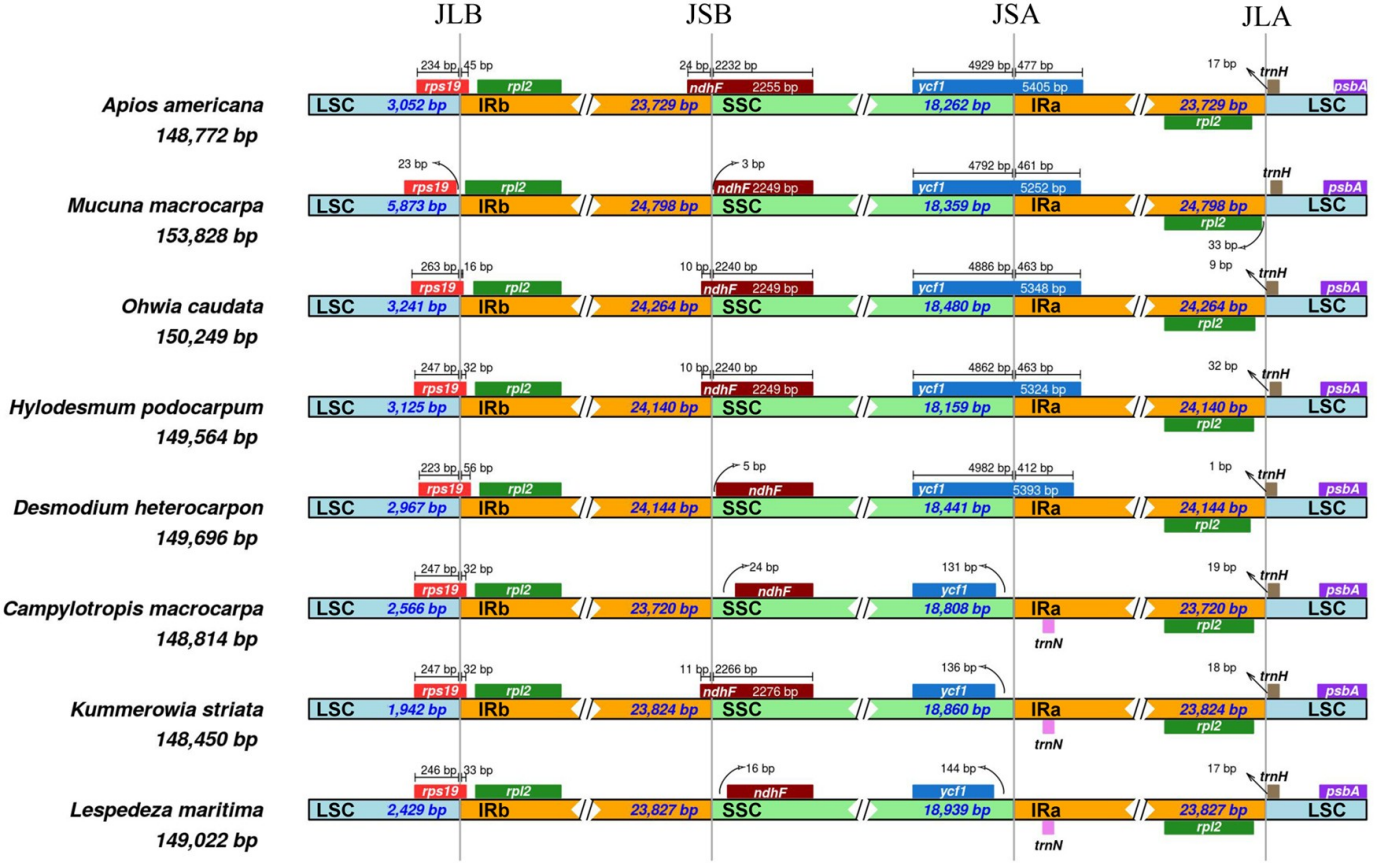
Comparison of junctions in plastid genomes sequenced here. JLA, junction IRa/LSC; JLB, junction IRb/LSC; JSA, junction IRa/SSC; JSB, junction IRb/SSC.

No severe expansion or contraction of IRs occurred at the junctions for three of four regions, i.e., JLA (junction IRa/LSC), JLB (junction IRb/LSC), and JSB (junction IRb/SSC) (Fig 4). The JLA, JLB, and JSB junctions were conserved as the *rps19*-*rpl2*, *rpl2*-*trnH*, and *ndhF* regions, respectively. However, we noted a distinction from JSA (junction IRa/SSC) for Desmodieae subtribe Lespedezinae (*Campylotropis*, *Kummerowia*, and *Lespedeza*). These genera were characterized by an approximately 600-bp contraction of IR that resulted from the absence of a partial *ycf1* copy (Table 1).

### Phylogenetic analyses

**Fig 5:**
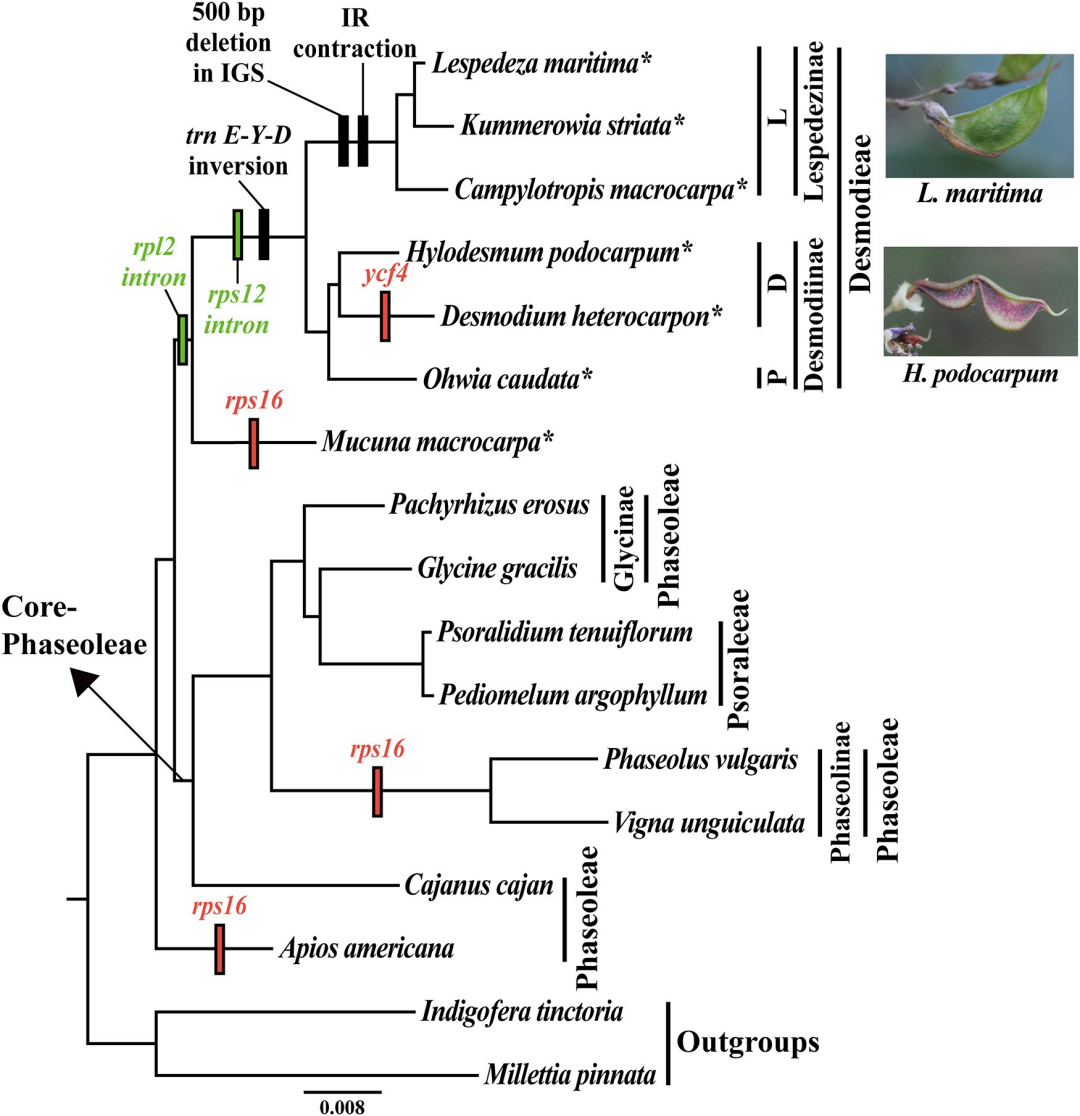
Maximum likelihood (ML) analysis of 17 legumes (15 Phaseoloid legumes and two outgroups) based on 67 concatenated protein-coding genes. This tree shown is the one of 1,000 trees derived from ML analysis of 67 concatenated protein-coding gene sequences, log likelihood number = -146379.564971. All nodes were supported with absolute 100% bootstrap values. The fruits of the subtribes [Desmodiinae (multiple-seeded loments) and Lespedezinae (single-seeded legume)] are shown on the side. Asterisks, taxa sequenced here; red and green rectangles on bar, intron and gene losses, respectively, from plastid genome; black rectangles, genome rearrangement events; D, DESMODIUM group; L, LESPEDEZA group; P, PHYLLODIUM group; IRLC, inverted repeat-lacking clade.

The cladistic (maximum likelihood) analyses of the aligned gene sequences revealed the phylogenetic relationships (Fig 5). The alignments included 50,301 nucleotide positions. All nodes of the ML tree were strongly supported by bootstrap values of 100%. On this ML tree, *Apios americana* is the sister group to the remaining Phaseoloid legumes. The remaining taxa were grouped into two clades. One clade consists of seven taxa from the tribe Desmodieae and its putative sister genus *Mucuna*, which was newly sequenced in the present study. The other clade is constructed of core-Phaseoleae members (except for *A*. *americana* and *M*. *macrocarpa*). In the former clade, two subtribes of Desmodieae (Desmodiinae and Lespedezinae) were also monophyletic groups. This topology of Desmodiinae coincided with the groups recognized within that subtribe: the DESMODIUM group (incl. *Desmodium* and *Hylodesmum*) and the PHYLLODIUM group (incl. *Ohwia*). In the case of Lespedezinae, *Lespedeza* and *Kummerowia* were more closely related to each other than to *Campylotropis*. In another clade, *Cajanus cajan* (L.) Huth (subtribe Cajaninae of Phaseoleae) was the earliest diverging taxon, while *Phaseolus vulgaris* and *Vigna unguiculata* (L.) Walp. (subtribe Phaseolinae of Phaseoleae) were reconstructed as a monophyletic group. In addition, *Pediomelum argophyllum* (Pursh) J.W. Grimes and *Psoralidium tenuiflorum* (Pursh) Rydb. (tribe Psoraleeae) were also a monophyletic group, nested within members of subtribe Glycininae of Phaseoleae [*Glycine gracilis* Skvortsov and *Pachyrhizus erosus* (L.) Urb.].

## Discussion

### Genome features

We sequenced seven plastomes that included species from six representative genera from the tribe Desmodieae and one species of *Mucuna*, the sister genus of the tribe in the previous supertrees [1–2]. The sizes of those seven plastomes do not deviate significantly from the typical genome length of Phaseoloids (e.g. *Glycine max*) [48]. One feature in common to all of these tested species is the absence of *infA* and *rpl22* like other legume species. However, two genes show signs of pseudogenization: *rps16* (in *Mucuna*) and *ycf4* (in *Desmodium*). A multiple gene-loss event of *rps16* has already been reported for various legume lineages [29, 35, 49]. Our comparative analysis demonstrated that the multiple loss event of *rps16* was also found in Phaseoloid legumes (Fig 5). The cause of such events is assumed to be a consequence of dual targeting of the nuclear *rps16* copy to the plastid as well as the mitochondria [50].

Results from an earlier study using slot blot hybridization [29] suggested a multiple loss of *ycf4* from numerous legume tribes. Moreover, Bailey et al. [8] have argued that the lack of *ycf4* can be used as molecular markers for the tribe Desmodieae, but they also note the extremely complex results from the subtribe Desmodiinae and Lespedezinae. In our results, Desmodieae plastomes showed substantial sequence substitutions and indels from each other (S3 Fig), which can produce complex signals from slot blot hybridization [8, 29]. However, sequence variations in most species are without internal stop codons or frameshift changes, with the exception of *D*. *heterocarpon*. Thus, the loss of *ycf4* is not a shared character of Desmodieae in Phaseoloid legumes. However, Magee et al. [37] identified hypermutation of *ycf4*, and associated multiple losses of IRLC and several Phaseoloid species. Hence, the sequence divergence and subsequent loss of *ycf4* in Desmodieae might not be caused by recent events in the tribe but may be related to ancient events that predated at least Phaseoloid, or the combination of IRLC and Phaseoloid.

Our complete plastome data shows the loss of an intron from *rpl2* and *rps12*. The former has previously been reported from three distantly related genera (*Bauhinia* L., *Soemmeringia* Mart., and *Mucuna*) and the tribe Desmodieae [8, 29, 51]. Recently, the *rpl2* intron loss from genus *Bauhinia* has been confirmed in a plastome analysis involving subfamily Cercidoideae [32]. Here, we confirmed that an intron of *rpl2* has been deleted from genus *Mucuna* and all six of the investigated Desmodieae genera.

The intron loss of *rps12* among legumes was first investigated by Jansen et al. [19] who determined it occurred independently in some genera of Desmodieae (*Desmodium*, *Kummerowia*, *Lespedeza*) and in most of the members of IRLC. The exceptions were some early-diverging genera such as *Callerya* Endl. and *Wisteria* Nutt. [19]. However, no examination had been made about whether the loss of the *rps12* intron is shared by Desmodieae and *Mucuna*. Our data indicated that the loss of the *rps12* intron was only limited to the six genera of Desmodieae, but not *Mucuna*. When considering our data, the *rpl2* intron loss, shared by Desmodieae and *Mucuna*, appears to have happened prior to the *rps12* intron loss since the tribe diverged from the genus.

### Desmodieae-specific inversion of the *trnE*-*Y*-*D* region and IR contraction in the subtribe Lespedezinae

We found a novel plastome rearrangement specific to tribe Desmodieae (Fig 3) that features an inversion approximately 1.5 kb long and contains three tRNA genes (*trnE*, *trnY*, and *trnD*). This inversion is located between *trnT*-*GGU* and *psbM*. The gene order from *trnT* through *trnD* (*trnT*-*GGU*—*trnE*-*UUC*—*trnY*-*GUA*—*trnD*-*GUC*) is well-conserved among seed plants. Moreover, this region (also known as the *trnD*-*trnT* intergenic spacer) is part of the “Tier 1 region” selected by Shaw et al. [52] for phylogenetic analysis because of its high potential as an informative character. Therefore, this region is frequently used as a molecular marker in various seed plants phylogenies, e.g., *Camassia* Lindl. [53] and *Pinus* L. [54], and with Phaseoloid legumes such as *Pueraria* DC. [55]. However, the disruption of this molecular marker (*trnD*-*trnT*) by inversion in Desmodieae means it is impossible to make a direct application of this marker to that tribe. As an alternative, future phylogenetic research on Phaseoloid legumes (including Desmodieae) would utilize only the conserved collinear block of *trnD*-*trnE*.

The plastome rearrangement can emerge through recombination [34, 56–57]. The Desmodieae-specific inversion discovered here coincides with a pair of 11-bp inverted repeats, “TATTGGATTTG” and “CAAATCCAATA” (Fig 3). If we consider this match of inversion breakpoints and inverted repeats, then we should regard the inversion of Desmodieae as a consequence of microhomology-driven recombination events via 11-bp inverted repeats. On the other hand, this inversion could be additional genetic evidence for the monophyly of Desmodieae. We found it interesting that one of the 11-bp repeat motifs is deleted from three genera of Desmodieae subtribe Lespedezinae (Fig 3). This was caused by a 500-bp deletion from the IGS between *trnT*-*GGU* and *psbM*. Variations in IGS length according to subtribes could serve as genetic markers for identifying the two subtribes of Desmodieae.

We detected an IR contraction from the SSC region in members of tribe Desmodieae subtribe Lespedezinae (*Campylotropis*, *Kummerowia*, and *Lespedeza*). Such variations in IR junctions from the LSC and SSC due to IR expansion or contraction have often been described from various types of land plants (e.g. [58–60]). In the plastomes of legumes, examples include notable IR variations such as a deletion in the IRLC [61] or expansion in the inverted repeat-expanding clade [62–63]. The contraction of IR shown from Lespedezinae is relatively small (ca. 600 bp) when comparing to other changes in the family [32]. Though, the removal of a partial copy of *ycf1* from the IR is noteworthy because the partial or entire duplication of *ycf1* by IRa is a well-conserved character among legumes [48, 64]. Therefore, the absence of a partial *ycf1* is a distinct feature of the plastomes for Desmodieae subtribe Lespedezinae.

### Phylogeny of the tribe Desmodieae within Phaseoloid legumes

The Maximum Likelihood (ML) analysis of 15 Phaseoloids legumes also used the related taxa *Millettia pinnata* (L.) Panigrahi and *Indigofera tinctoria* L. as outgroups. Based upon 67 conserved protein coding-genes, a *matK* phylogeny analysis revealed these outgroups as sister groups [2]. Earlier plastome-scale phylogenetic analyses of Fabaceae members had also been conducted by our research group as well as others (e.g., [35–36]). However, the work presented here is the first to focus on Phaseoloids and to cover tribe Desmodieae and *Mucuna*. In doing so, we compared the plastome phylogeny of Phaseoloids with previous phylogenies based on partial DNAs (e.g., [1–3, 10, 12, 55]) and found that our results are consistent with the earlier findings, even though our sampling was not as complete as the earlier studies. As shown by our plastome phylogenetic tree (Fig 5), *Apios* is earliest branching while the remaining Phaseoloid legumes form two clades: core-Phaseoleae, a group that includes three subtribes (Cajaninae, Glycininae, and Phaseolinae) and the tribe Psoraleeae [4], and Desmodieae/*Mucuna*. In the former, Phaseolinae is confirmed as a monophyletic group, and Psoraleeae is also a monophyletic group that is nested within Glycininae, which is supported by previous phylogenetic studies [2, 55]. Members of Desmodieae are gathered as a monophyletic group and as a sister to *Mucuna*. Consequently, Desmodieae and Psoraleeae are found to monophyletic while simultaneously being included within Phaseoleae. Given this, we might reconsider the taxonomic rank of Desmodieae and Psoraleeae as a subtribe of Phaseoleae or we might change the other subtribes of Phaseoleae to a tribal level [1]. This particular topology may also explain the loss of genes/introns among Phaseoloid legumes. As shown in our plastome maps (Fig 2 and S1 Fig), the *rpl2* intron loss is shared in Desmodieae and *Mucuna* [8, 29] while the *rps12* intron loss is detected only in Desmodieae [19]. Taking into account this plastome phylogeny, it is likely that the *rpl2* intron loss preceded the *rps12* intron loss within the Phaseoloids. Furthermore, we might infer that the *rps16* gene loss occurred independently at least three times in those Phaseoloids, i.e., two losses when *Apios* and *Mucuna* were separated from their recent ancestors and a third loss when subtribe Phaseolinae diverged.

The two subtribes of Desmodieae (Desmodiinae and Lespedezinae) are well-supported as a monophyletic group, an outcome similar to that already reported from phylogenetic studies of that tribe (e.g., [12]). This finding is also in accordance with circumstances based on morphological traits [7]. Desmodiinae plants bear multiple-seeded loments, standard petal without auricles at the base, and frequent stipels whereas Lespedezinae has single-seeded fruits, a standard with auricles at the base, and no stipels [7]. These novel genome rearrangements that were determined for Lespedezinae in the present study (i.e., 500-bp deletion in the IGS at *trnE*-*psbM*, and an IR contraction; Figs 3 and 4) support the monophyly of Lespedezinae. In addition, our phylogenetic tree recognized the three groups (DESMODIUM, PHYLLODIUM, and LESPEDEZA) of this tribe that are suggested by *rbcL* phylogeny and morphological traits [5]. One taxonomic study based on *rbcL* phylogeny and morphological characters placed *Hylodesmum* within DESMODIUM [5]; however, *matK* phylogenetic tree placed *Hylodesmum* at early branching group of Desmodiinae [2]. Our ML tree shows that *H*. *podocarpum* is grouped with *Desmodium heterocarpon* (included in DESMODIUM group), similar to another cpDNA (*rbcL*, *psbA*-*trnH*) phylogeny [12]. Because of controversy regarding the relationship among genera of Lespedezinae based on morphological characters, several phylogenetic studies have been conducted (e.g., [16–17]). Our plastome tree indicated that *Campylotropis* is a sister to the group containing *Kummerowia* and *Lespedeza*, and this result coincides with those from other analyses. Therefore, we recognize that *Lespedeza* is more closely related to *Kummerowia*.

## Supporting information

S1 Fig Maps of plastid genome of tribe Desmodieae and genus *Mucuna*. (A) *Hylodesmum podocarpum* subsp. *podocarpum*. (B) *Ohwia caudata*. (C) *Campylotropis macrocarpa*. (D) *Kummerowia striata*. (E) *Lespedeza maritima*. (F) *Mucuna macrocarpa*. Genes on outside of outer circle are transcribed in clockwise direction; those on inside of outer circle are transcribed in counterclockwise direction. Colored rectangles indicate functional genes, with categories shown on bottom left. Gray scale in inner circle indicates GC content of plastid genome.
(TIF)

S2 Fig Comparison of *rpl2* and *rps12* introns in Phaseoloid legumes. Red-shaded rectangles indicate absence of introns. Parts of introns were omitted. (A) *rpl2* sequence. (B) *rps12* sequence.
(TIF)

S3 Fig Alignment of *ycf4* gene sequences in Phaseoloid legumes. Red-shaded rectangles indicate severe nucleotide variations that resulted in frameshift or missing start codon. This gene from *Desmodium heterocarpon* is considered to be pseudogenes.
(TIF)

S4 Fig Alignment of *rps16* gene sequences in Phaseoloid legumes. Red-shaded rectangles indicate severe nucleotide variations that resulted in frameshift or missing start codon. Parts of introns were omitted. *rps16* genes from *Apios americana*, *Phaseolus vulgaris*, and *Vigna radiata* are considered to be pseudogenes.
(TIF)

S1 Table Sampling and sequencing information for taxa from tribe Desmodieae and *Mucuna*. (PDF)

S2 Table GenBank accession numbers of taxon used in plastome phylogeny. (PDF)

S3 Table Gene list employed in plastome phylogeny. (PDF)

S4 Table Gene list of taxa analyzed from tribe Desmodieae and *Mucuna*. (PDF)

S1 Fasta File Fasta file of 67 concatenated protein-coding genes used in Maximum likelihood analysis. (FASTA)
