# Supplementary figures and images for: Plastid genome evolution in tribe Desmodieae (Fabaceae: Papilionoideae)
Source: PLoS One. 2019 Jun 24;14(6):e0218743. doi: 10.1371/journal.pone.0218743 (PMC6590825; doi:10.1371/journal.pone.0218743)

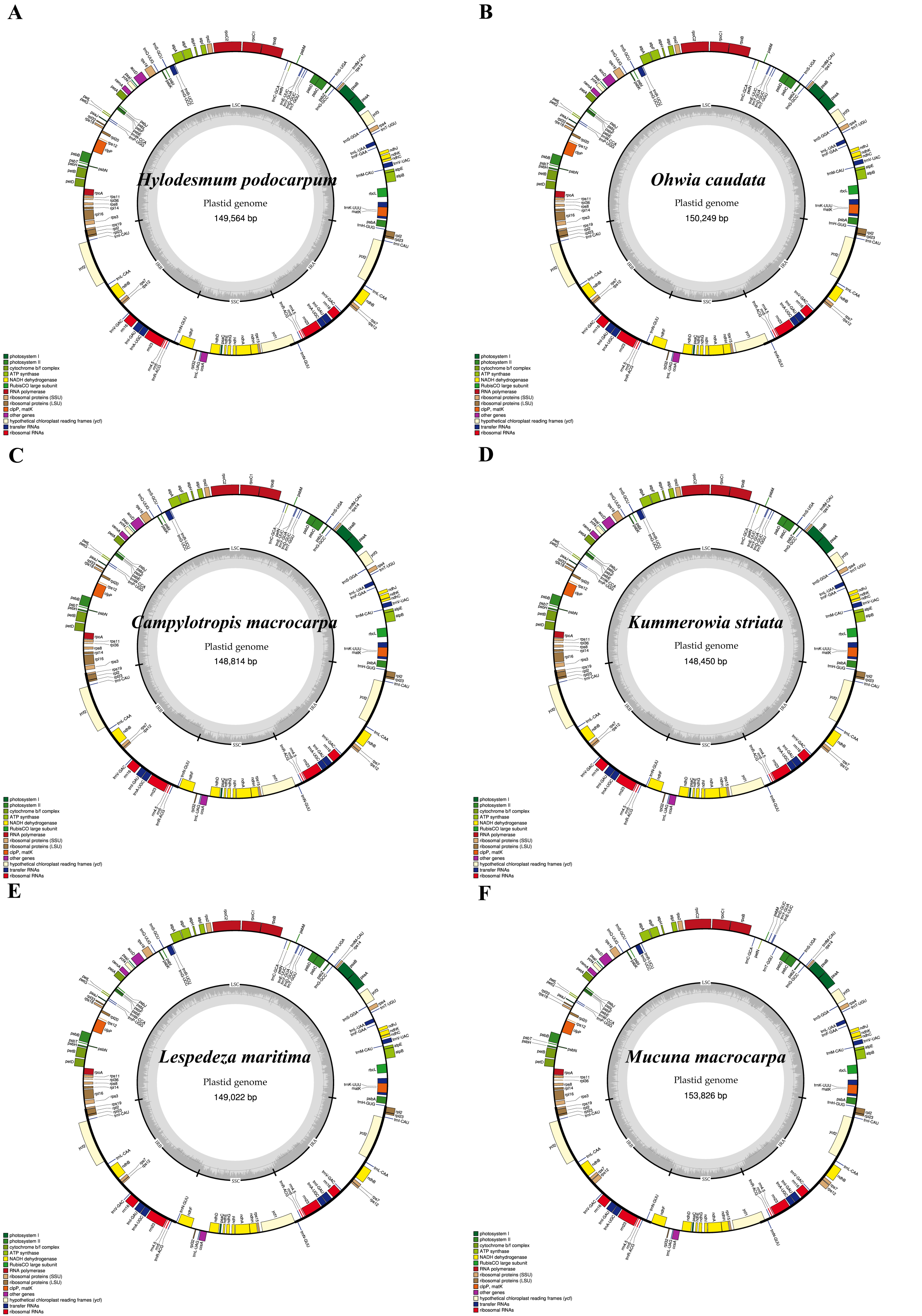

Supplement: S1 Fig — (A) Hylodesmum podocarpum subsp. podocarpum. (B) Ohwia caudata. (C) Campylotropis macrocarpa. (D) Kummerowia striata. (E) Lespedeza maritima. (F) Mucuna macrocarpa. Genes on outside of outer circle are transcribed in clockwise direction; those on inside of outer circle are transcribed in counterclockwise direction. Colored rectangles indicate functional genes, with categories shown on bottom left. Gray scale in inner circle indicates GC content of plastid genome. (TIF) [file pone.0218743.s001.tif]

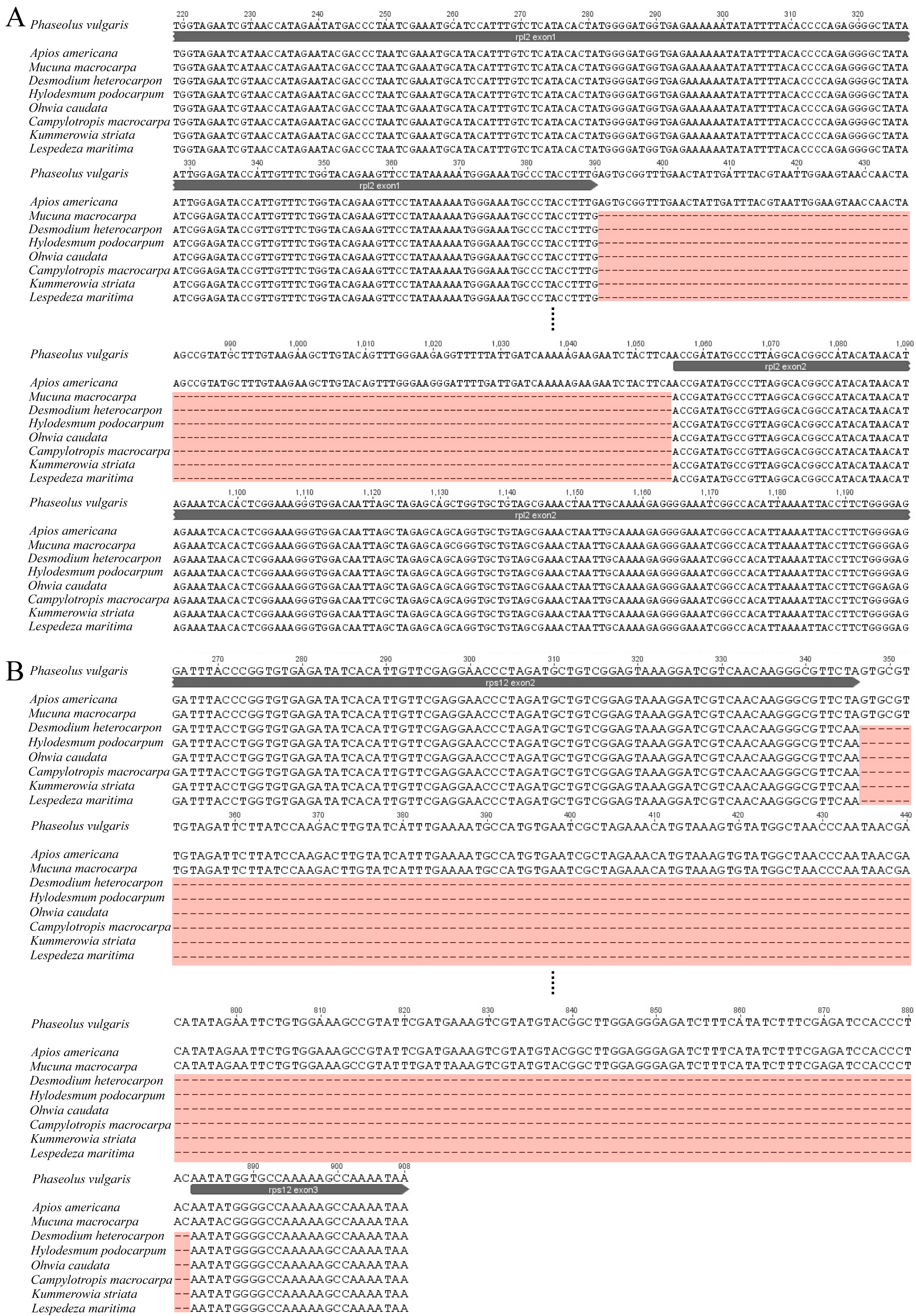

Supplement: S2 Fig — Red-shaded rectangles indicate absence of introns. Parts of introns were omitted. (A) rpl2 sequence. (B) rps12 sequence. (TIF) [file pone.0218743.s002.tif]

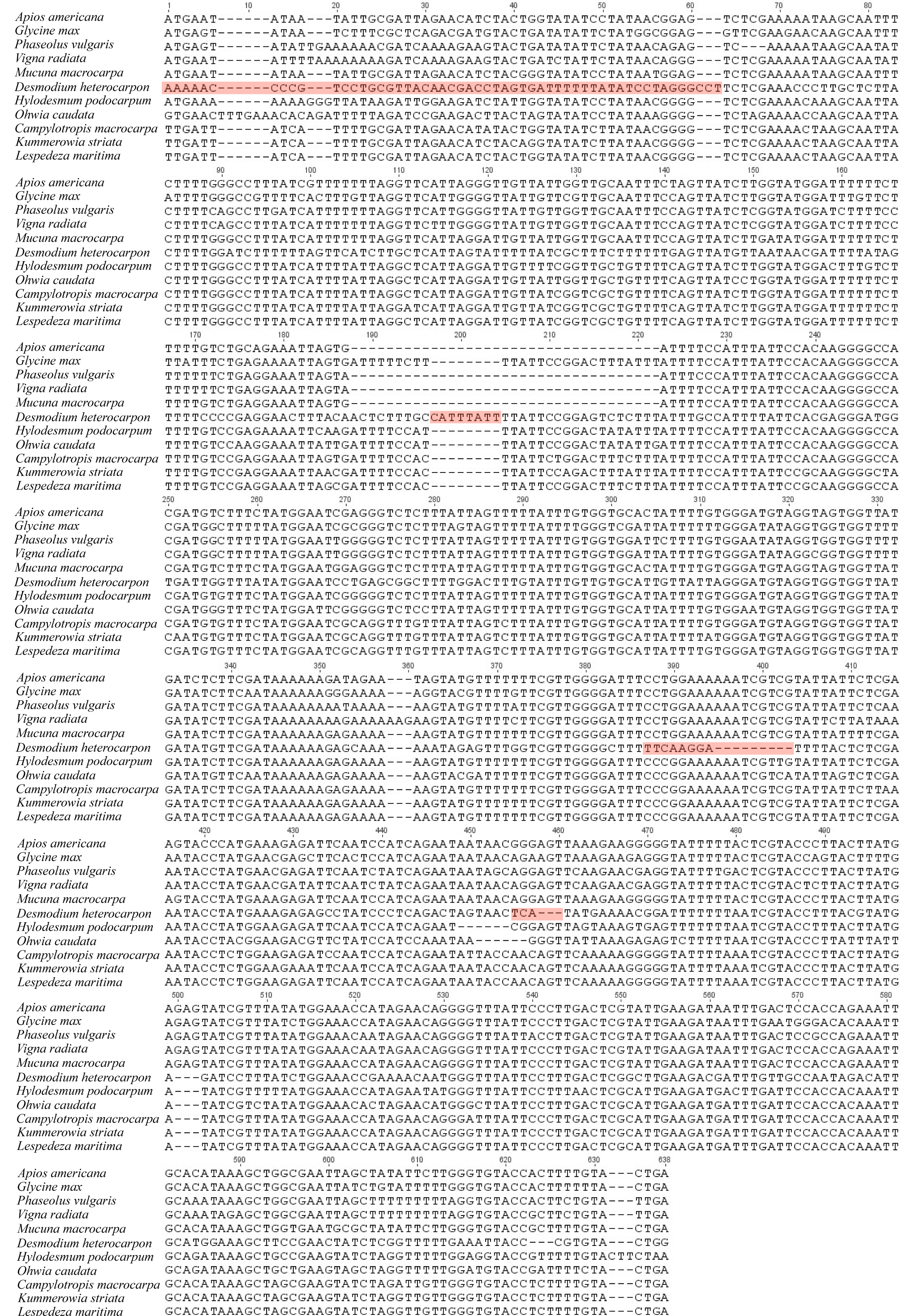

Supplement: S3 Fig — Red-shaded rectangles indicate severe nucleotide variations that resulted in frameshift or missing start codon. This gene from Desmodium heterocarpon is considered to be pseudogenes. (TIF) [file pone.0218743.s003.tif]

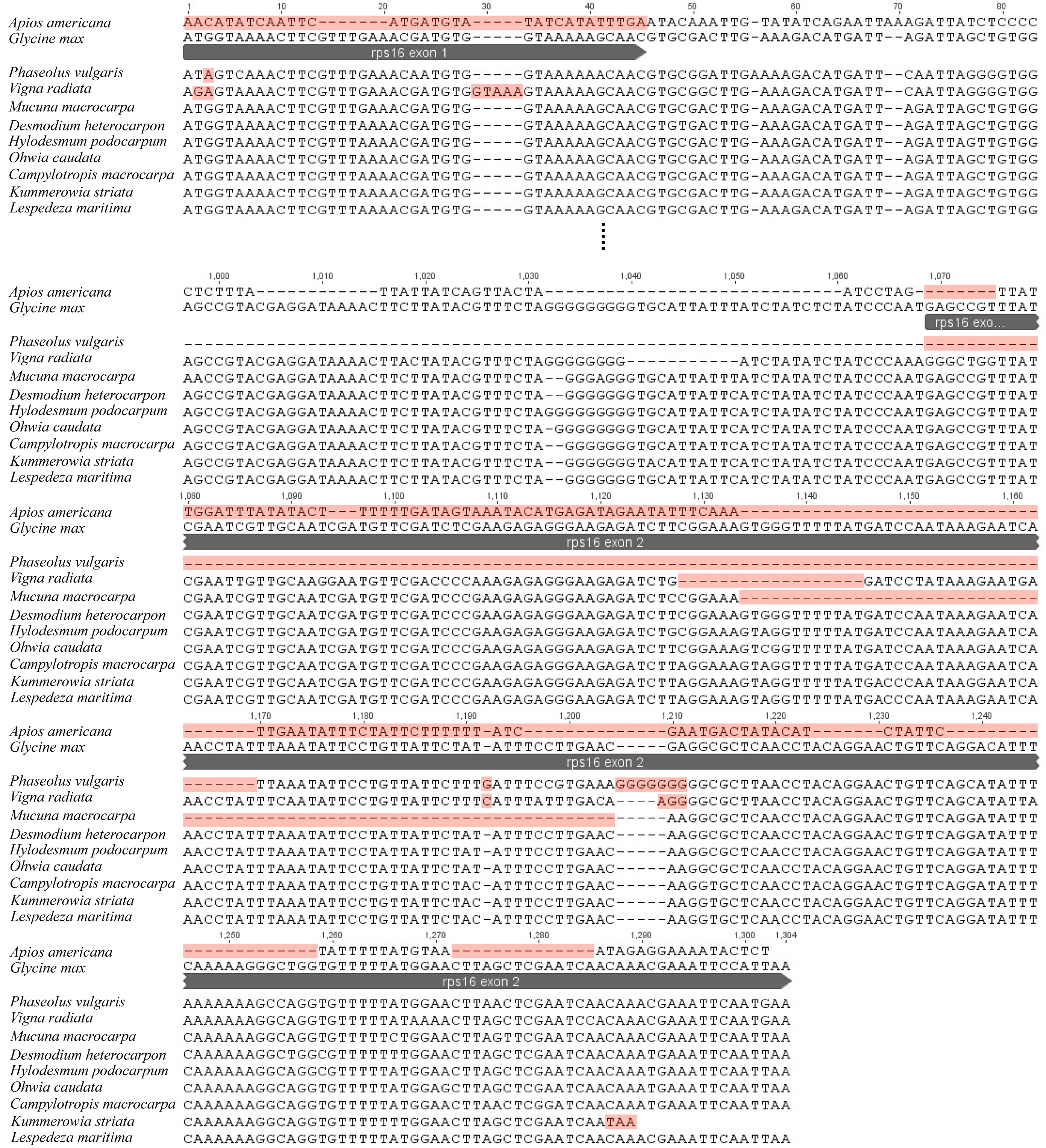

Supplement: S4 Fig — Red-shaded rectangles indicate severe nucleotide variations that resulted in frameshift or missing start codon. Parts of introns were omitted. rps16 genes from Apios americana, Phaseolus vulgaris, and Vigna radiata are considered to be pseudogenes. (TIF) [file pone.0218743.s004.tif]
