# Supplementary material for: Plastid genome evolution in tribe Desmodieae (Fabaceae: Papilionoideae)
Source: PLoS One. 2019 Jun 24;14(6):e0218743. doi: 10.1371/journal.pone.0218743 (PMC6590825; doi:10.1371/journal.pone.0218743)
Supplement: S1 Table — (PDF) [file pone.0218743.s005.pdf]

S1 Table. Sampling and sequencing information for taxa from tribe Desmodieae and *Mucuna*.

Species	Locality	Coordinate	Voucher no.	Produced paired reads
Campylotropis macrocarpa	Mt. Hwanghak, Chilgok-gun, Gyeongsangbuk-do, Korea	36°01′58″N, 128°30′33″E	109901	2,982,620
Desmodium heterocarpon	Sallokdoro, Seogwipo-si, Jeju-do, Korea	33°18′45″N, 126°35′20″E	98555	3,144,944
Hylodesmum podocarpum	Isl. Oeyeon, Boryeong-si, Chungcheongnam-do, Korea	36°13′41″N, 126°05′03″E	169505	3,104,242
Kummerowia striata	Mt. Geomdan, Gwangju-si, Gyeonggi-do, Korea	37°27′28″N, 127°11′23″E	DP167901	3,125,556
Lespedeza maritima	Peak Gyeokja, Bogil-myeon, Wando-gun, Jeollanam-do, Korea	34°08′16″N, 126°31′49″E	DP149121	10,758,454
Mucuna macrocarpa	Kunigami, Okinawa, Japan	26°44′40″N, 128°10′51″E	15001	3,279,328
Ohwia caudata	Jeju-do, Korea	33°31′94″N, 126°42′16″E	NIBR378625	3,321,814
