# Supplementary material for: Plastid genome evolution in tribe Desmodieae (Fabaceae: Papilionoideae)
Source: PLoS One. 2019 Jun 24;14(6):e0218743. doi: 10.1371/journal.pone.0218743 (PMC6590825; doi:10.1371/journal.pone.0218743)
Supplement: S2 Table — (PDF) [file pone.0218743.s006.pdf]

S2 Table. GenBank accession numbers of taxon used in plastome phylogeny.

Species	Accession no.
Outgroups	
Indigofera tinctoria	KJ468098
Millettia pinnata	JN673818
Tribe Phaseoleae	
Apios americana	KF856618
Cajanus cajan	KX672004
Glycine gracilis	KX029327
Pachyrhizus erosus	KJ468100
Phaseolus vulgaris	EU196765
Vigna unguiculata	JQ755301
Tribe Psoraleeae	
Pediomelum argophyllum	KP126866
Psoralidium tenuiflorum	KP126859

Note: Sequence data of *Pediomelum argophyllum* and *Psoralidium tenuiflorum* (tribe Psoraleeae) is provided as the cluster of 83 genes.
