# Supplementary material for: Plastid genome evolution in tribe Desmodieae (Fabaceae: Papilionoideae)
Source: PLoS One. 2019 Jun 24;14(6):e0218743. doi: 10.1371/journal.pone.0218743 (PMC6590825; doi:10.1371/journal.pone.0218743)
Supplement: S3 Table — (PDF) [file pone.0218743.s007.pdf]

S3 Table. Gene list employed in plastome phylogeny.

Name of gene
atpA, *atpB*, *atpE*, *atpF*, *atpH*, *atpI*, *ccsA*, *cemA*, *clpP*, *matK*, *ndhA*, *ndhB*, *ndhC*, *ndhD*, *ndhE*, *ndhF*, *ndhG*, *ndhH*, *ndhI*, *ndhJ*, *ndhK*, *petA*, *petB*, *petD*, *petG*, *petL*, *psaA*, *psaB*, *psaC*, *psaI*, *psaJ*, *psbA*, *psbB*, *psbC*, *psbD*, *psbE*, *psbF*, *psbH*, *psbI*, *psbJ*, *psbK*, *psbL*, *psbM*, *psbN*, *psbT*, *psbZ*, *rbcL*, *rpl2*, *rpl14*, *rpl16*, *rpl20*, *rpl23*, *rpl36*, *rpoA*, *rpoB*, *rpoC1*, *rpoC2*, *rps2*, *rps3*, *rps4*, *rps7*, *rps8*, *rps11*, *rps14*, *rps15*, *rps19*, *ycf3*
