# Supplementary material for: Plastid genome evolution in tribe Desmodieae (Fabaceae: Papilionoideae)
Source: PLoS One. 2019 Jun 24;14(6):e0218743. doi: 10.1371/journal.pone.0218743 (PMC6590825; doi:10.1371/journal.pone.0218743)
Supplement: S4 Table — (PDF) [file pone.0218743.s008.pdf]

S4 Table. Gene list of taxa analyzed from tribe Desmodieae and *Mucuna*.

	Group of gene	Name of gene*	No.
RNA genes	Ribosomal RNAs	*rrn4.5*(x2), *rrn5*(x2), *rrn16*(x2), *rrn23*(x2)	8
	Transfer RNAs	*trnA-UGC*[a](x2), *trnC-GCA*, *trnD-GUC*, *trnE-UUC*, *trnF-GAA*, *trnfM-CAU*, *trnG-GCC*, *trnG-UCC*[a], *trnH-GUG*, *trnI-CAU*(x2), *trnI-GAU*(x2), *trnK-UUU*[a], *trnL-CAA*(x2), *trnL-UAA*[a], *trnL-UAG*, *trnM-CAU*, *trnN-GUU*(x2), *trnP-UGG*, *trnQ-UUG*, *trnR-ACG*(x2), *trnR-UCU*, *trnS-GCU*, *trnS-GGA*, *trnS-UGA*, *trnT-GGU*, *trnT-UGU*, *trnV-GAC*(x2), *trnV-UAC*[a], *trnW-CCA*, *trnY-GUA*	33
Protein genes	Photosynthesis		
	Photosystem I	*psaA*, *psaB*, *psaC*, *psaI*, *psaJ*	5
	Photosystem II	*psbA*, *psbB*, *psbC*, *psbD*, *psbE*, *psbF*, *psbH*, *psbI*, *psbJ*, *psbK*, *psbL*, *psbM*, *psbN*, *psbT*, *psbZ*	15
	Cytochrome	*petA*, *petB*[a], *petD*[a], *petG*, *petL*, *petN*	6
	ATP synthase	*atpA*, *atpB*, *atpE*, *atpF*[a], *atpH*, *atpI*	6
	Rubisco	*rbcL*	1
	NADH dehydrogenease	*ndhA*[a], *ndhB*[a](x2), *ndhC*, *ndhD*, *ndhE*, *ndhF*, *ndhG*, *ndhH*, *ndhI*, *ndhJ*, *ndhK*	12
	ATP-dependent protease subunit P	*clpP*[a]	1
	Chloroplast envelope membrane protein	*cemA*	1
Ribosomal proteins	large units	*rpl2*(x2), *rpl14*, *rpl16*[a], *rpl20*, *rpl23*(x2), *rpl32*, *rpl33*, *rpl36*	10
	small units	*rps2*, *rps3*, *rps4*, *rps7*(x2), *rps8*, *rps11*, *rps12*[a](x2), *rps14*, *rps15*, *rps16*[a], *rps18*, *rps19*	14
Transcription /translation	RNA polymerase	*rpoA*, *rpoB*, *rpoC1*[a], *rpoC2*	4
	Miscellaneous proteins	*accD*, *ccsA*, *matK*	3
	Hypothetical proteins & Conserved reading frame	*ycf1*, *ycf2*(x2), *ycf3*[a], *ycf4*	5
Total			128

Note: *ycf4* and *rps16* are pseudo gene in *Desmodium heterocarpon* and *Mucuna macrocarpa*, respectively.

*(x2): duplicated genes, [a]: genes having introns, ψ: duplicated segment,
